# Helping Others Helps Me: Prosocial Behavior and Satisfaction With Life During the COVID-19 Pandemic

**DOI:** 10.3389/fpsyg.2022.762445

**Published:** 2022-01-27

**Authors:** Juan C. Espinosa, Concha Antón, Merlin Patricia Grueso Hinestroza

**Affiliations:** ^1^School of Business and Management, Universidad del Rosario, Bogotá, Colombia; ^2^Department of Social Psychology and Anthropology, Universidad de Salamanca, Salamanca, Spain

**Keywords:** prosocial behaviors, negative emotions, satisfaction with life, COVID-19, Colombia

## Abstract

Prosocial behavior (PsB) and its effects have been analyzed in times of crisis and natural disasters, although never before in the face of such exceptional circumstances as those created by the COVID-19 pandemic. This research analyzes the role of PsB on satisfaction with life (SWL) in Colombia, considering the negative emotional impact of events that began in February 2020. We conduct an exploratory analysis using a sample of Colombia’s general population (*N* = 2,574; 53.2% women) with an average age of 44.66 years (*SD* = 15.36). Using the Classification Tree technique, we find that engaging in one or more PsBs (e.g., donating money or sharing food) enhances SWL and decreases the impact of negative emotions such as pessimism, indecisiveness, and irritability that have emerged during the COVID-19 pandemic. These findings are significant because they confirm the importance of life satisfaction as a personal resource for coping with complex situations and provide evidence of the benefits of PsB on one’s wellbeing.

## Introduction

The COVID-19 pandemic has severely impacted societies (Alkhamshi et al., 2021; Coccia, 2021), businesses (Singh et al., 2021), and personal finances (Cudris-Torres et al., 2021). This unprecedented situation has restricted productivity in many sectors, negatively impacting economies (Cudris-Torres et al., 2021) and posing challenges to people everywhere (Tse et al., 2021). During times of crises such as the COVID-19 pandemic as well as natural disasters like earthquakes and hurricanes (Maki et al., 2019), prosociality or prosocial behavior (PsB) has been of interest to researchers. Zagefka (2021) tested the predictors of people’s intentions to make donations to help those suffering from crises created by the COVID-19 pandemic in the United Kingdom (U.K.) and concluded that perceiving a common fate in the context of a crisis was associated with PsB. The evidence also reveals that PsB exerts positive effects on the helpers, decreasing their stress and loneliness and increasing social connection, solidarity, happiness, and shared resilience (Zaki, 2020).

The COVID-19 pandemic has affected the quality of life for people around the world (Cudris-Torres et al., 2021; Zakeri et al., 2021), and Satisfaction With Life (SWL) as a measure of quality of life has been impacted in various ways (e.g., Clench-Aas and Holte, 2017; Cudris-Torres et al., 2021; Hermassi et al., 2021; Yıldırım and Arslan, 2021). For example, Hermassi et al. (2021) demonstrated that SWL decreased during COVID-19 lockdowns in Qatar. Clench-Aas and Holte (2017) obtained similar results in analyzing the effects of the 2008 global financial crisis in several European countries. They showed that the 2008 financial crisis affected life satisfaction for the populations studied at two levels. Life satisfaction declined sharply in the short term, and showed a progressively milder decline over the long term, adding to the strong negative relationship between SWL and unemployment that lasted for several years. This led them to conclude that after the onset of the 2008 financial crisis a long-term decline in life satisfaction tended to occur, especially in higher socio-economic groups.

The negative effects of the COVID-19 pandemic are now being studied, and there is evidence that it has taken a heavy toll on the mental health of people around the world who are experiencing a deteriorating sense of wellbeing and high levels of stress (Israelashvili, 2021). A study of 405 undergraduate and postgraduate students in China found that while quarantined at home, nearly half of university students were prone to experience negative emotions such as anxiety (42.2%), stress (29.4%), and depression (44.0%) (Han et al., 2021). Similarly, a 2020 study of 170 nurses in China found their most widespread emotion was anxiety, with 30% reporting that they felt anxious (Sun et al., 2021).

Research on the psychological factors or processes that could buffer the negative impact of a crisis such as the COVID-19 pandemic on mental health and related negative behaviors that may persist over a long period is scant (Chong et al., 2021). In particular, we note the importance of understanding the impact of PsB on mental health in the context of the COVID-19 pandemic, as it differs from human-caused disasters (Maki et al., 2019). Because much of the research on PsB have been conducted during times of security and stability, there is little knowledge about the impact of PsBs during a global pandemic (Yue and Yang, 2021). In fact, some authors point out that the potentially devastating effects of the Covid19 pandemic can be mitigated by the development of studies from the social and behavioral sciences (Van Bavel et al., 2020).

Nonetheless, studies developed in the context of the COVID-19 pandemic have demonstrated that PsB can be manifested in different ways, depending on the cultural context (Tse et al., 2021). This is of great relevance for the context of this study (i.e., the urban population in Colombia) because previous studies on SWL concluded that research participants experienced satisfaction despite the financial crisis created by the COVID-19 pandemic (Cudris-Torres et al., 2021). Therefore, this study seeks to address the following research question: How do PsBs improve SWL, considering the negative emotions that emerged during the COVID-19 pandemic in the Colombian population?

The structure of this research is organized as follows. Section “Theoretical Framework” describes our theoretical framework and develops our research hypotheses. Section “Materials and Methods” describes the methodology used; section “Results” presents the results; and section “Discussion” discusses. Section “Conclusion” offers conclusions, limitations of the study, and suggestions for future research.

## Theoretical Framework

### Prosocial Behavior During the COVID-19 Pandemic

PsB is a broad category of behaviors that “attend to the benefit or welfare of another person(s)” (Burks and Kobus, 2012, p. 319), and focuses on “observable actions that benefit others regardless of whether there are costs to the helper or issues such as self-sacrifice” (Burks and Kobus, 2012, p. 319). That is, PsB is conceptualized in a way that focuses on the helping act and its positive benefits for both the recipient and the helper, i.e., feeling good about helping someone or helping someone to feel good (Burks and Kobus, 2012).

According to previous research, in a threatening situation such as the COVID-19 pandemic both prosocial and self-interested behaviors can emerge (Tse et al., 2021). In a cross-cultural study that includes 251 participants from the United Kingdom, 268 from the United States (U.S.), 197 from Germany (DE), and 200 from Hong Kong (HK), Tse et al. (2021) found that threat perception is positively associated with prosocial acts and hoarding. They also observed cross-cultural differences, wherein the effects were stronger in more individualistic countries (U.K., U.S.) than in the less individualistic ones (HK, DE). PsB can be explained from the Social Identity Model of Collective Behavior in emergencies and disasters, developed from research conducted over many years and presented in Drury (2018), which points out that people display solidarity when they have a shared social identity. According to this social identity model, facing a common crisis such as the COVID-19 pandemic leads people to develop an emerging sense of community and group identification (Ntontis et al., 2021).

Based on the aforementioned, the following hypothesis is formulated (H1): In the context of a COVID-19 health emergency, most people perform Prosocial Behaviors.

### Negative Emotions During the COVID-19 Pandemic

Negative emotions have also been studied in times of crisis. In a systematic literature review, Brooks et al. (2020) showed that pandemic-induced quarantines had negative psychological effects on individuals. The outbreak of COVID-19 has been linked with adverse psychological outcomes (Alkhamshi et al., 2021; Han et al., 2021; Sui et al., 2021; Sun et al., 2021). For example, a cross-sectional study by Abed Alah et al. (2021) conducted via an internet-based survey in November and December 2020 found that 12.4, 14.2, and 18.5% of the participants reported symptoms of depression, anxiety, and stress, respectively. Alkhamshi et al. (2021) reported much higher levels of depression (50.25%), anxiety (50%), and loss of confidence (35.5%) in their study of participants from Saudi society. Han et al. (2021) found that participants in their study, undergraduate and postgraduate Chinese students, were prone to negative emotions such as stress, anxiety, and depression related to the pandemic. Sui et al. (2021) found that 24.9% of a sampling of nurses in China (*n* = 339) experienced negative emotions due to the pandemic. Finally, Sun et al. (2021) used a sample of 202 nurses at the Wuhan Makeshift Hospital and concluded that among the negative emotions experienced by the participants, anxiety was the most prominent.

Based on the above, the following hypothesis is formulated (H2): In the context of COVID-19 health emergency, Negative Emotions are present in the population.

### Satisfaction With Life During the COVID-19 Pandemic

According to Diener et al. (1985), SWL refers to a “cognitive, judgmental process” (p. 71) and is considered a subjective component of wellbeing. SWL can therefore be defined as a global cognitive judgment about one’s life, where the evaluation is not based on objective parameters but rather depends on a comparison of life circumstances with one’s own internal patterns, mediated by the cultural context in which one lives (Vinaccia Alpi et al., 2019). Given its explanatory power in terms of human behavior, SWL has received attention in the context of crises. Clench-Aas and Holte (2017) found a sharp decrease in SWL at the beginning of the 2008 global financial crisis and another, but a less severe decrease in SWL in 2011. A decline in SWL after the 2008 financial crisis was especially evident among those in higher occupational levels and in highly educated groups. Hermassi et al. (2021) concluded that COVID-19 confinement decreased SWL as well as physical activity in Qatar. Finally, Yıldırım and Arslan (2021) demonstrated that changes in individuals’ SWL are related to the suffering caused by the COVID-19 pandemic.

In contrast with recent findings regarding the psychological effects of the COVID-19 pandemic, Cudris-Torres et al. (2021) study individuals in Colombia and find no relationship between financial hardships resulting from COVID-19 and SWL, although 66% of the study’s participants do not have a source of income other than their main occupation; 42% consider their economic situation to have worsened compared with previous years; and 39% have not been meeting their financial obligations promptly during their pandemic-induced confinement.

Based on the above, the following hypothesis is formulated (H3): In the context of COVID-19 health emergency, Satisfaction with Life has decreased.

### The Relationship Between Prosocial Behavior and Satisfaction With Life

In the context of an emergency PsB can contribute to survival, wellbeing (Drury, 2018), life satisfaction, and psychological flourishing (Miles et al., 2021) and may play a significant role in mitigating the adverse effects of COVID-19 on mental health (Chong et al., 2021). Using a sample size of 514 Hong Kong citizens, Chong et al. (2021) found that psychological flexibility and PsB may significantly mitigate the adverse effects of COVID-19 and its perceived threat to public mental health. Recent research reveals that PsB has a positive effect on helpers, increasing happiness and decreasing loneliness and stress (Zaki, 2020). There is also evidence that PsB increases positive collective outcomes such as solidarity, social connection, and shared resilience (Zaki, 2020). Finally, a growing body of research demonstrates that PsB such as volunteer work both improves the individual’s wellbeing and helps the wider community (Hansen et al., 2018).

### The Relationship Between Prosocial Behavior and Negative Emotions

Early in the COVID-19 crisis, there were concerns about social engagement (McGuire et al., 2020). A U.S.-based online survey was used to examine patterns of social support and PsB for people with and without depression or anxiety. The results evince that PsB was negatively associated with anxiety. Similarly, Ferguson and Masser (2018) found that PsB can be used to manage negative emotions and enhance wellbeing.

### The Relationship Between Negative Emotions and Satisfaction With Life

Several studies demonstrate that psychological disorders are significantly associated with poor/no SWL (Zakeri et al., 2021). Özer et al. (2021) determined that the perceived risk of COVID-19 increases death anxiety and decreases individuals’ psychological wellbeing and SWL. A study conducted using two representative samples in the United States and United Kingdom (*N* = 2,000) during the first wave of the COVID-19 pandemic concluded that positive emotions are particularly important to mental health in the context of increased levels of negative emotional experiences (Israelashvili, 2021).

Despite research on PsB, negative emotions, and general life satisfaction, few studies have analyzed the protective effect of PsB in crises, such as the current COVID-19 pandemic. Similarly, although studies have investigated the factors that explain SWL in other contexts, in Colombia such studies are scarce, with results that are contrary to findings obtained in other countries. Therefore, this study explores how PsB improves SWL in the context of the negative emotions that have emerged among the Colombian population during the COVID-19 pandemic.

Based on the aforementioned the following hypothesis is formulated (H4): In the COVID-19 health emergency context, PsB reduces the impact of negative emotions on Satisfaction with Life.

## Materials and Methods

### Data

We designed a survey-based study using a quantitative approach and a non-experimental, cross-sectional dataset. The research sample comprises 2,574 adults (53.2% women) from the general population in 10 cities in Colombia, with an average age of 44.66 years (*SD* = 15.85). A multistage sampling by internet and telephone communication was performed. Table 1 presents the distribution of participants by age ranges.

**TABLE 1:** Participants by age range.

Age	Frequency	Percent	Cumulative percent
18–25	376	14.6	14.6
26–35	515	20.0	34.6
36–45	446	17.3	51.9
46–55	487	18.9	70.9
56–65	419	16.3	87.1
>65	331	12.9	100.0
Total	2,574	100.0	

*Authors.*

Three questionnaires were used in this study: a PsB scale, a Negative Emotions Checklist, and an SWL Scale (SWLS).

#### Prosocial Behavior Scale

PsB was examined using six true/false questions (see Table 2). The reliability of the scale was adequate (Cronbach’s alpha = 0.68). An exploratory principal component factor analysis revealed a two-factor structure that explains 60.21% of the observed variance [Kaiser Meyer Olkin (KMO) = 0.72]. The first factor (40.38% of total variance explained) aggregates items related to help provided through NGOs, whereas the second factor (19.82% of total variance explained) contains items related to helping provided directly (person-to-person). Details are presented in Table 2.

**TABLE 2:** Prosocial behavior items and factor loading.

	Factor loading	
Items	C.1	C.2
Give food to families in need.		0.83
Give money or products to friends.		0.77
Give money to NGOs for humanitarian aid.	0.71	
Give food to the neediest people.		0.64
Give money or products to health professionals.	0.84	
Give money or food to NGOs for animal protection.	0.76	

*N, 2,574.*

*Authors.*

#### Negative Emotions Checklist

A checklist (Yes or No responses) of 10 negative emotions experienced by participants during quarantine was used. The reliability of the checklist was satisfactory (Cronbach’s alpha = 0.81). An exploratory principal component factor analysis revealed a unidimensional factor structure that explains 38.08% of the observed variance (KMO = 0.89). Details are presented in Table 3.

**TABLE 3:** Negative emotions checklist items and factor loading.

Checklist items	Factor loading
Downheartedness	0.65
Pessimism	0.54
Failure	0.54
Dissatisfaction	0.65
Desire to cry	0.69
Irritability	0.66
Disinterested in everything	0.62
Indecision	0.63
Loss of appetite	0.56
Fatigue	0.62

*N, 2,574.*

*Authors.*

#### Satisfaction With Life Scale

The SWLS is a widely used measure of subjective wellbeing (Bamer et al., 2021). It consists of a five-item questionnaire using a Likert scale developed by Diener et al. (1985) and adapted for Colombia by Vinaccia Alpi et al. (2019). In this study, the SWLS shows satisfactory reliability (Cronbach’s Alpha = 0.89). The exploratory principal component factor analysis yielded a unidimensional factor structure that explains 69.23% of the observed variance (KMO = 0.88). Details are presented in Table 4.

**TABLE 4:** Satisfaction with life scale items and factor loading.

Items	Factor loading
In most ways my life is close to my ideal	0.81
The conditions of my life are excellent	0.85
I am satisfied with my life	0.86
So far, I have gotten the important things I want in life	0.78
If I could live my life over, I would change almost nothing	0.86

*N, 2,433.*

*Authors.*

### Procedures

Information was provided by the participants voluntarily, without any type of incentive. The respondents were invited to participate in the study; confidentiality of the data was guaranteed; and participants were told the analyses would be carried out globally. The questionnaires were completed during the months of July and August 2020. Once the information was captured, descriptive, psychometric, and inferential analyses were performed. A Decision Tree procedure was used, which generates a classification model that identifies subsets of values of a dependent variable (criterion) based on the values of independent variables (predictors). The procedure can be used for exploratory or confirmatory purposes, depending on whether there is a previous classification of individuals. In the present study, the procedure was used for exploratory purposes and included SWL as a criterion variable and Negative Emotions and PsB as predictors.

## Results

Descriptive statistics for our three variables, PsBs, SWL, and Negative Emotions, are presented in Table 5. The SWLS displays a moderate average, whereas Negative Emotions and PsB have low averages.

**TABLE 5:** Descriptive statistics.

	Min	Max	Mean	*SD*	*t*	*P*
Direct prosocial behavior (C2)*^a^*	0	3	1.10	1.10	51.00	0.00
Indirect prosocial behavior (C1)*^a^*	0	3	0.27	0.67	20.23	0.00
Negative emotions*^a^*	0	10	1.52	2.16	35.60	0.00
SWLS*^b^*	5	35	24.22	5.79	−26.31	0.00

*N, 2,574.*

*^a^Test value, 0.*

*^b^Test value, 27.31 (Vinaccia Alpi et al., 2019).*

*Authors.*

Firstly, the Colombian showed a Direct and Indirect PsB (1.10 and 0.27, respectively). Based on these findings, Hypothesis 1 was empirically tested in this study (Table 5). Furthermore, a significant difference was found between the Direct and Indirect PsB (*t* = 39.82, *p* = 0.00), indicating that Colombians preferred to engage in direct prosocial actions during the pandemic (60%) and little in indirect prosocial behaviors (17%).

**FIGURE 1:**
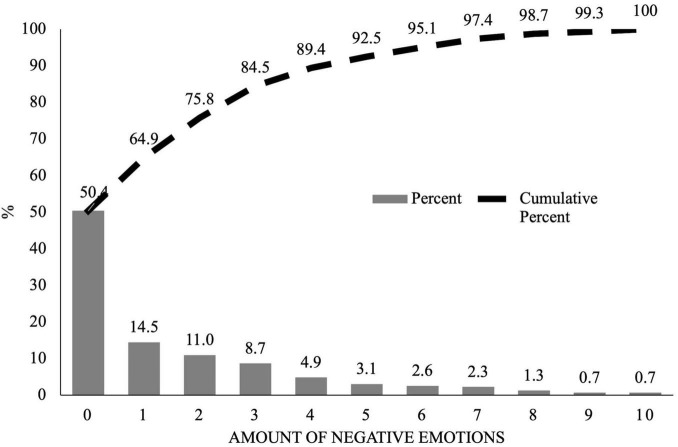
Distribution of negative emotions.

Examining the number of Negative Emotions (Figure 1) reveals that half of the participants did not report any of the 10 negative emotions specified and that a quarter of the participants reported only one or two negative emotions. The remaining 25% of respondents reported between three and 10 negative emotions, and 5% reported seven or more negative emotions. The top 10% presented five or more. Based on these findings, Hypothesis 2 was empirically tested in this study, that is, a significant presence of negative emotions in the Colombian population.

**FIGURE 2:**
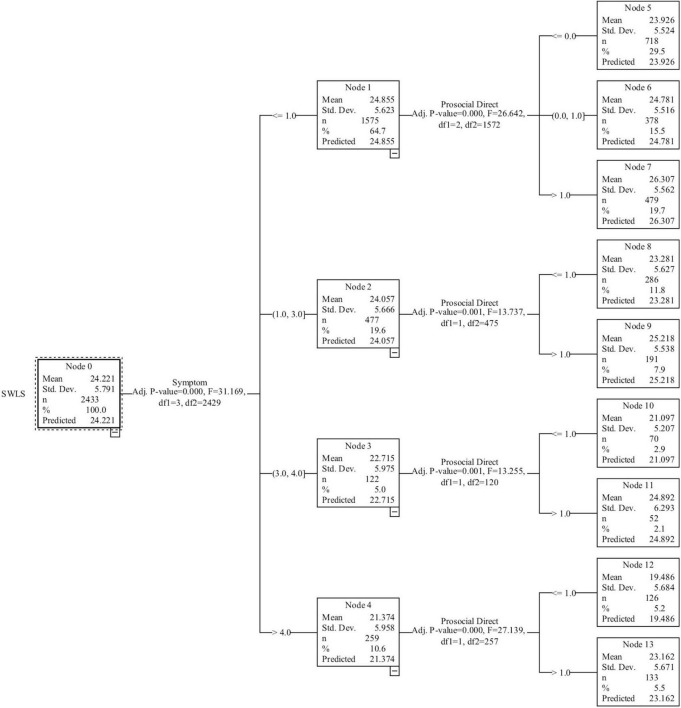
Classification tree of SWLS based on negative emotions and direct PsBs.

Afterward, we identify nine SWL nodes based on the number of Negative Emotions and Direct PsBs. The Indirect PsBs were not useful for the classification tree (Figure 2). The group used in the classification procedure consisted of 2,433 individuals with a mean of 24.22 (Node 0) on the SWLS. This first value represents the mean on SWLS for this study and was contrasted with a reference value for the Colombian population (Vinaccia Alpi et al., 2019). Based on these findings, Hypothesis 3 was empirically tested in this study (Table 5). Based on the number of Negative Emotions, four groups are distinguished that differ significantly from each other (*F* = 31.17, *p* = 0.00).

The first node aggregates approximately 65% of the participants who are characterized by having reported no negative emotions (mean SWLS = 24.86). The second node combines roughly 20% of respondents who report one to three negative emotions (mean SWLS = 24.06). The third node is made up of the 5% of participants who report between three and four negative emotions (mean SWLS = 22.72). The fourth node is made up of roughly 11% of respondents who reported more than four negative emotions (mean SWLS = 21.37). These first four nodes allow us to conclude that negative emotions are more common among those who report a lower SWL. It is possible that people who had a lower SWL before the pandemic were more vulnerable to the negative emotions generated by the crisis.

At the second level of the tree, Direct PsBs (DPsBs) allow for a more precise classification based on whether the participants report no DPsBs or report that they performed at least one of the behaviors described in the survey (Figure 2). From the first node, nodes 5, 6, and 7 emerge (*F* = 26.64, *p* = 0.00), whereas nodes 8 and 9 emerge from the second node (*F* = 13.74, *p* = 0.00). Nodes 10 and 11 emerge from the third node (*F* = 13.26, *p* = 0.00). Finally, the fourth node splits into nodes 12 and 13 (*F* = 27.14, *p* = 0.00). A consistent finding across all of the child nodes is that when people engage in PsBs their SWL is higher, even among those with fairly low SWL, which according to the first node corresponds to those who have more negative emotions also.

In sum, as Figure 2 demonstrates, the highest average SWL is seen in node 7, which aggregates approximately 20% of the participants who reported no negative emotions and performed DPsBs (mean SWLS = 26.31). In contrast, the lowest average SWL is found in node 12, which aggregates approximately 5% of the participants and is characterized by not engaging in any DPsBs and presenting more than four negative emotions (mean SWLS = 19.49). Based on these findings, Hypothesis 4 was empirically tested in this study.

## Discussion

The academic literature evinces that PsB is related to happiness (Sinyavskaya et al., 2019), wellbeing (Drury, 2018), psychological flourishing, and SWL (Miles et al., 2021). There is also evidence, although scarce, that PsB is significantly mitigated the adverse effects of the COVID-19 pandemic on individuals’ mental health (Chong et al., 2021), as it has been shown that the pandemic has had a significant effect on individuals’ experience of negative emotions (Abed Alah et al., 2021; Alkhamshi et al., 2021; Han et al., 2021; Sui et al., 2021; Sun et al., 2021), evidenced through an increase in levels of depression (Abed Alah et al., 2021; Alkhamshi et al., 2021; Han et al., 2021), anxiety (Abed Alah et al., 2021; Alkhamshi et al., 2021; Han et al., 2021; Sun et al., 2021), stress (Abed Alah et al., 2021; Han et al., 2021), and decreased self-confidence (Alkhamshi et al., 2021).

In the face of the challenging and unprecedented situation presented by the COVID-19 pandemic (Tse et al., 2021), our research explores whether PsB moderates the effect of negative emotions on SWL through a survey of 2,574 people in 10 Colombian cities. Based on the data gathered through the survey, our results reveal that SWL was affect by COVID-19 pandemic are in line with Hermassi et al. (2021); Israelashvili (2021), and Yıldırım and Arslan (2021). However, it is important to notice that SWL maintains at a moderate level which can be explained in light of Vinaccia Alpi et al. (2019) who point out that SWL is a subjective perception that is greatly affected by cultural factors. Colombia has faced an internal armed conflict for more than five decades (Grueso Hinestroza et al., 2019) that has not yet been entirely resolved. Therefore, it is understandable that Colombia’s citizens have developed a capacity to face challenging situations without a serious negative impact on their perceived quality of life. In fact, even in the midst of the acute conflict the country faced prior to signing the peace agreement with the former FARC guerrillas, Colombia reported high levels of happiness between 2010 and 2012 (ranked 35th out of 156 countries) and between 2015 and 2018 (ranked 37th out of 156) (Helliwell et al., 2018).

The results discussed above lead us to conclude that in general, Colombians possess emotional resources that allow them to cope with the negative consequences of the pandemic. This may imply that in the near future Colombians may face a greater emotional weariness from the pandemic that may require psychosocial attention, especially because the country’s “safety net” for the neediest within its population is deficient, and those responsibilities are assumed by a social support network composed of family and friends.

When societies face a threat from health crises or natural disasters, PsB is relevant because it not only benefits those who receive help but also benefits the giver (Yue and Yang, 2021). Two types of PsBs were identified in this study: *DPsB* that involves actions with immediate, local impact and *IPsB* that has a more removed, less immediate impact. Most participants in our research reported engaging in *DPsB* rather than going through intermediaries, such as foundations that are related to *IPsB*. As Politi et al. (2021) stated, people are generally more inclined to support and help those who are physically and psychologically close (relatives, neighbors, and fellow citizens) than those who are physically and psychologically distant (acquaintances, foreigners, and strangers).

The fact that study participants displayed more DPsBs is also explained by a contextual factor. The people of Colombia perceive a high degree of corruption in their country and therefore prefer to act in ways that directly help those most in need. According to the Corruption Perception Index 2020 (Transparency International, 2021), Colombia scores 39 out of a possible 100, where 0 means very high corruption, and 100 is an absence of corruption, ranking 92nd out of 180 countries. This lack of trust in institutions can be a determining factor in the choice of how to engage in actions that benefit others when faced with crises.

PsB is expected in times of crisis, especially when a large percentage of the human population is affected. The social identity model explains that when societies are faced with a common fate, such as the COVID-19 pandemic, people develop a stronger sense of community and identification with the group (Ntontis et al., 2021). In our research behaviors such us giving food to families, money to friends, and support to the neediest can be understood from the social identity model.

Finally, our research demonstrates that engaging in DPsB protects one’s SWL in times of crisis when negative emotions may emerge. In this sense, our study makes a contribution by recognizing that DPsB could reduce the negative impact of the COVID-19 pandemic on mental health particularly in terms of SWL. Understanding PsB’s impact on mental health in the context of the COVID-19 pandemic is a relevant contribution because this relationship has been explained more frequently in contexts of human-caused disasters (Maki et al., 2019). As Yue and Yang (2021) noted, understanding PsB during a global pandemic is an important addition to the literature because much of the research on this topic has been conducted during times of security and stability.

## Conclusion

Our research results allow us to conclude that helping others helps people to increase their own SWL. We find that while SWL is inversely related to the presence of negative emotions, the impact of these negative feelings is reduced by engaging in PsBs. This finding is significant because it provides evidence of the personal benefit (in terms of personal life satisfaction) of behaviors aimed at improving the wellbeing of others. Additionally, our study identifies that among those who report greater SWL and are therefore less vulnerable to negative emotions, engaging in PsB has a potentiating effect, increasing one’s level of life satisfaction. A clear recommendation emerges from this suppressor/potentiating effect of PsB on life satisfaction as a function of negative feelings: PsB is a social mechanism that favors SWL and makes it possible to cope collectively with moments of crisis and high demands that can threaten the segments of a population that are facing the crisis.

It is important to point out some limitations of this study. First, the data were obtained in 10 main cities of the country and did not consider participants from rural communities. It is possible that there are differences in the perception of life satisfaction among individuals living in cities compared with those living in rural areas, and it is possible that the relationships between the variables studied may differ as a function of this attribute. In addition, due to the restrictions imposed by the COVID-19 pandemic, interviews were conducted by telephone rather than in person, which could have affected the quality of the responses. Furthermore, we relied on self-reported data to measure SWL, negative emotions, and PsBs. Although this approach is common in social science research, there may be concerns with social desirability or inaccurate estimation. Finally, the study is exploratory in scope with a correlational design, which does not allow the results to be generalized.

Topics for future research in this area could include studies of specific population groups defined by occupation, such as health care personnel, hospitality industry workers, or teachers. Comparative studies could be developed between people living in urban centers and in rural areas. Other research could employ confirmatory analysis techniques, such as structural equation modeling.

## Data Availability Statement

The raw data supporting the conclusions of this article will be made available by the authors, without undue reservation.

## Ethics Statement

Ethical review and approval were not required for the study on human participants in accordance with the local legislation and institutional requirements. Written informed consent for participation was not required for this study in accordance with the national legislation and the institutional requirements.

## Author Contributions

All authors listed have made a substantial, direct, and intellectual contribution to the work, and approved it for publication.

## Conflict of Interest

The authors declare that the research was conducted in the absence of any commercial or financial relationships that could be construed as a potential conflict of interest.

## Publisher’s Note

All claims expressed in this article are solely those of the authors and do not necessarily represent those of their affiliated organizations, or those of the publisher, the editors and the reviewers. Any product that may be evaluated in this article, or claim that may be made by its manufacturer, is not guaranteed or endorsed by the publisher.
